# TRPM6 acts as a prognostic biomarker and mediates Mg^2+^ dependent tumor suppression in colon adenocarcinoma

**DOI:** 10.3389/fimmu.2025.1686461

**Published:** 2026-01-05

**Authors:** Lichao Cao, Yuxin Ren, Xinru Yu, Sihan Wang, Hezi Zhang, Erfei Chen

**Affiliations:** 1 School of Medicine, Northwest University, Xi’an, China; 2 Department of Research and Development, Shenzhen Nucleus Gene Technology Co., Ltd., Shenzhen, China; 3 Key Laboratory of Resource Biology and Biotechnology in Western China, Ministry of Education, Northwest University, Xi’an, China; 4 Shaanxi Provincial Key Laboratory of Infection and Immune Diseases, Shaanxi Provincial People’s Hospital, Xi’an, China

**Keywords:** colon adenocarcinoma, transient receptor potential melastatin channel family genes, magnesium, pan-cancer, prognosis

## Abstract

**Introduction:**

Colon adenocarcinoma (COAD) is a malignant neoplasm derived from colonic epithelial cells with poor prognosis in advanced stages. This study aimed to identify critical molecular regulators of COAD and develop robust biomarkers for prognosis and therapy.

**Methods:**

Transcriptome data from public databases were analyzed: differential expression of TRPM family genes in COAD and pan-cancer pathogenic associations of TRPM6 were assessed via the Wilcoxon test, with analyses of consensus molecular subtypes, clinical relevance, and survival also performed. TRPM6-regulated differentially expressed genes (DEGs) were identified for functional enrichment, and immune/tumor microenvironment correlations were evaluated using the Spearman test. *In vitro*, HCT116 and SW480 cells were transfected with TRPM6 siRNAs, with proliferation (CCK-8), migration (Transwell), and marker expression (RT-qPCR) assessed.

**Results and Discussion:**

The study showed TRPM family genes, particularly TRPM4 and TRPM6, had differential expression across COAD subtypes/stages, with high expression correlating with favorable prognosis. TRPM6 regulates cancers via neural pathways, associates with immune/tumor microenvironments, and has pan-cancer diagnostic value. TRPM6 knockdown promoted colon cancer cell proliferation and migration, while concurrent TRPM6 knockdown and high Mg^2+^ treatment attenuated Mg^2+^-mediated tumor suppression. These findings highlight TRPM6 as a pivotal mediator of Mg^2+^ functions and a valuable candidate for COAD prognostic classification and therapeutic intervention, providing a foundation for future studies on its regulatory roles in tumor progression and Mg^2+^-based therapies.

## Introduction

Colon adenocarcinoma (COAD), a malignancy arising from colonic epithelial cells, represents the predominant subtype of colon cancer (CRC) (1). In recent years, it has emerged as a major public health burden in China, characterized by increasing incidence and mortality rates (2), with over 380,000 new colorectal cancer cases projected annually (3). Established risk factors for COAD include environmental and genetic influences, excessive alcohol consumption, tobacco use, dietary intake of red or processed meats, and lack of physical activity (4). While surgical resection remains the primary curative modality, often complemented by chemotherapy and radiotherapy (5), the prognosis for advanced COAD remains poor, with a 5-year survival rate of only ~10% among patients with distant metastases. Despite advancements in immunotherapy and targeted therapies, unmet needs persist in improving outcomes for these patients (6). Consequently, identifying key molecular regulators of COAD and developing robust biomarkers for prognosis and treatment selection represent critical priorities in contemporary oncology research.

The transient receptor potential melastatin (TRPM) channel family comprises eight intrinsic plasma membrane ion channels (TRPM1-TRPM8) with conserved structural features, including N-terminal, transmembrane, and C-terminal domains, that are widely expressed across human tissues (7, 8). Emerging evidence highlights their dual roles in oncogenesis, with members exhibiting both pro- and anti-tumorigenic functions across diverse cancer types (8, 9). Recent studies have found that TRPM4 is abnormally expressed in some CRC samples (10), but its association with COAD molecular subtypes (such as CMS), clinical stage, and regulatory mechanism are still unclear. Notably, magnesium (Mg^2+^) homeostasis, linked to reduced CRC risk via dietary intake (11), as a magnesium ion channel, TRPM6 is widely distributed in the epithelial cells of the intestine and kidneys, which also plays a significant role in the formation of CRC. Existing studies suggest that Mg^2+^ can regulate intracellular Mg^2+^ concentration through TRPM6, thereby affecting DNA repair enzyme activity or metabolic pathways—key processes that influence tumor cell survival and proliferation (12). In CRC tissues, TRPM6 mRNA is downregulated, and its protein expression correlates positively with overall survival (OS) (13), while murine and human tissue studies confirm its robust expression in intestinal epithelia and renal distal convoluted tubules (14). TRPM6 exerts intricate and multifaceted effects in regulating the infiltration and functional status of pivotal immune cells within the tumor stroma. For example, TRPM6 is expressed in tumor-associated macrophages (TAMs) and modulates their phagocytic function and pro-inflammatory phenotype by participating in calcium signaling pathways (15). However, it remains unclear whether this TRPM6-mediated Mg^2+^ regulatory axis functions in COAD through similar mechanisms, nor has its role in modulating COAD-specific pathological processes been systematically explored.

Therefore, in this study, we first utilized the pan-cancer public database to investigate the differential expression of the TRPM family among pan-cancer sample groups, the differences in clinical characteristics subgroups, the differences in CMS subtypes, and its correlation with immune genes. Subsequently, this study represents the first report to uncover the regulatory mechanism underlying TRPM6-mediated magnesium ion (Mg^2+^) tumor suppression, which operates through the neural pathway-immune microenvironment axis. Furthermore, these findings identify TRPM6 as a novel potential therapeutic target, thereby providing a new theoretical basis and actionable direction for the optimization of immunotherapeutic strategies in COAD.

## Materials and methods

### Acquisition of data

In this research, the GSE39582 (Platform: GPL570) and GSE17536 (Platform: GPL570) for TRPM family genes were obtained from Gene Expression Omnibus (GEO) (https://www.ncbi.nlm.nih.gov/geo/) databases, where GSE39582 comprised 566 CRC samples along with 19 non-tumoral colorectal mucosas samples and GSE17536 contained 177 tumor samples. Upon the identification of the highly relevant with TRPM6 in COAD, the RNA sequencing information, clinical information, survival information of TCGA-COAD obtained from the UCSC Xena platform (https://xenabrowser.net/datapages/), which contained 459 tumor samples, along with 93 healthy control samples. Prior to differential expression analysis and subsequent functional analyses, all data were uniformly processed for batch effect correction and data normalization to eliminate technical heterogeneity in this study. Additionally, an integrated pan-cancer transcriptome sequencing dataset was obtained and analyzed by R package TCGAplot (v 8.0.0) (https://github.com/tjhwangxiong/TCGAplot).

### Analyzing differential expression

So as to explore the differential expression among TRPM family genes in COAD, the Wilcoxon test was utilized in GSE39582 (*P* < 0.05). Furthermore, in the quest to delineate the spectrum of cancers that manifest a profound association with TRPM6 at the pathogenic level in pan-cancer, we harnessed Wilcoxon test with the results visualized in a box plot by ggplot2 (v 3.4.1) package (*P* < 0.05).

### Consensus molecular subtypes and clinical relevance analysis

In order to provide a foundation for clinical stratification and subtype-specific targeted interventions with TRPM family genes, the CMS classification system categorizes CRC into four distinct subtypes according to RNA expression profiles (16), the Wilcoxon test method was utilized in GSE39582 and validation in GSE17536 (*P* < 0.05). To further explore the clinical relevance of TRPM family genes, we subsequently collected information on various levels for tumor M stage, N stage, T stage, and Stage, and investigated the expression differences among the Stage subgroups by Wilcoxon test (*P* < 0.05). Similarly, after stratifying the information of Age, Gender, Stage in TCGA-COAD, the Wilcoxon test method was employed to determine in which groups there were significant differences in gene expression levels of TRPM6, thereby assessing its clinical relevance (*P* < 0.05).

### Survival and prognostic analysis of TRPM family genes in COAD

Then the tumor samples were classified into high- and low-expression groups by the optimal cutoff value of the expression level of the TRPM family genes in GSE39582 and GSE17536. Then, between two expression groups, the survminer (v 0.4.9) package was utilized to generate Kaplan-Meier (KM) survival curves, and evaluated by plotting the receiver operating characteristic (ROC) curves with the use of timeROC (v 0.4) package for the reliability of TRPM family genes (area under curve (AUC) > 0.6).

### Exploration of the common regulatory mechanisms of TRPM6 in highly relevant cancer types

To explore the molecular regulatory mechanisms commonly mediated by TRPM6 across all highly relevant cancer types, we utilized the featureCount data from the transcriptome sequencing of these cancer cohorts. Tumor samples were stratified into high- and low-TRPM6 expression groups based on the median expression level of TRPM6. Subsequently, we conducted differential expression analysis between these groups using the edgeR (v 4.0.16) and limma package (v 3.58.1) to identify differentially expressed genes (DEGs). The thresholds for differential analysis were set with an absolute fold change greater than 1 and a p-value less than 0.05 and selected the genes that show differential expression in at least 5 types of cancer as the TRPM6-significantly regulated genes. The biological functions and signal pathways for significantly regulated genes were analyzed for Gene Ontology (GO) and Kyoto Encyclopedia of Genes and Genomes (KEGG) enrichment (false discovery rate [FDR] < 0.05) utilizing clusterProfiler (v 4.7.1.3). The results of top 10 GO and top 5 KEGG enrichment pathways were visualized in this study.

### Analysis of the immune microenvironment and tumor microenvironment

To explore the relationship between TRPM6 and immune - related indicators across pan-cancer, Spearman correlation analysis was employed to determine the correlations between TRPM6 and immune cells, immunostimulants, immunosuppressants, immune scores, immune checkpoints, and chemokines (|cor| > 0.1, *P* < 0.05). So as to explore the relationship between TRPM6 and tumor mutation burden (TMB) and microsatellite instability (MSI) across pan-cancer, R package TCGAplot (v 8.0.0) was also utilized in this study.

### Cell culture

Human colorectal cancer (CRC) cell lines HCT116 and SW480 were obtained from Wuhan Procell Life Science Technology Co., Ltd. HCT116 cells were cultured in McCoy’s 5A medium (PM150710B, Procell), and SW480 cells were cultured in RPMI-1640 medium (11875093, Gibco). Both cell lines were supplemented with 10% exosome-free fetal bovine serum (FBS; C04001-500, Vivacell) and maintained at 37 °C in a humidified incubator with 5% CO_2_.

### siRNA transfection

Transfection was performed using the reverse transfection method: The transfection complex prepared with jetPRIME^®^ transfection reagent (101000046, Polyplus) was incubated for 10–15 min, mixed with an appropriate volume of cell suspension (at 40%–60% confluency per well), and directly seeded into 12-well plates. The final concentration of siRNAs in SW480 and HCT116 cells was 50 nM. The sequence of the two siRNA targeting TRPM6 were detailed in **Table 1**.

**Table 1:** The specific primer sequences of the two siRNA targeting TRPM6.

Genes	Sequences 5’-3’
si-TRPM6#1-F	GAGCAUGGGUCAAAGCGAATT
si-TRPM6#1-R	UUCGCUUUGACCCAUGCUCCC
si-TRPM6#3-F	CGCUAUCGCUACAUCAUGATT
si-TRPM6#3-R	UCAUGAUGUAGCGAUAGCGGT

### CCK-8 assay

HCT116 cells and SW480 cells from both control and experimental groups were seeded in 96-well plates at densities of 1×10^4^ cells per well (HCT116) and 1.5×10^4^ cells per well (SW480), respectively, with five replicate wells per group. For HCT116 cells, CCK-8 reagent (C6005M, Uelandy, 10 μL per well) was added at 0, 12, 24, and 36 hours; for SW480 cells, measurements were taken at 0, 12, 24, 36, and 48 hours. After 1.5 hours of incubation at 37°C, absorbance at 450 nm was measured using a microplate reader (BioTek). The relative proliferation rate was calculated by normalizing the OD450 values of individual wells to the baseline (0 h for HCT116, 12 h for SW480) measurements of their respective groups.

### Transwell assay

Cell migration was assessed using a transwell chamber system. A 24-well transwell chamber with an 8-μm polycarbonate membrane (3422, Corning Inc) was used for the transmigration assay. Isolated cells were resuspended in serum-free medium, and 1×10^5^ suspended cells in 200 μL were carefully loaded into the upper chamber. The lower chamber was filled with medium containing 10% FBS as a chemoattractant. After 48 hours of incubation at 37°C with 5% CO_2_, the membrane was washed twice with ice-cold PBS. Cells were fixed with 0.4% formaldehyde for 10 minutes and stained with 0.1% crystal violet for 10 minutes. Non-migrated cells on the upper surface were gently removed during two PBS washes to eliminate non-adherent staining. Finally, migrated cells were quantified microscopically using image analysis software.

### Real-time quantitative polymerase chain reaction

Total RNA was extracted from cells using the Vazyme Super FastPure Cell RNA Isolation Kit (RC102-01, Vazyme) and quantified. Genomic DNA was removed by treating 2 μg of RNA with 4 μL of 4×gDNA wiper Mix in a single tube of a 200 μL 8-tube strip, adjusted to a final volume of 16 μL with RNase-free H_2_O. After thorough mixing, the mixture was incubated at 42°C for 2 minutes. Reverse transcription was performed by adding 4 μL of 5×HiScript II qRT SuperMix II (R223-01, Vazyme), followed by mixing and incubation at 50°C for 15 minutes and 85°C for 5 seconds. The resulting cDNA was diluted 10-fold with nuclease-free H_2_O and mixed well for immediate use in qPCR. GAPDH served as the internal reference gene, and relative gene expression was calculated using the 2^−ΔΔCt^ method. All specific primer sequences are listed in **Table 2**.

**Table 2:** The specific primer sequences of RT-qPCR.

Genes	Sequences 5’-3’
TRPM6-F	AGCACAATCATACCCAGCTCA
TRPM6-R	CATGGTCTCCAATCAGTCGGC
Ki-67-F	GCCTGCTCGACCCTACAGA
Ki-67-R	GCTTGTCAACTGCGGTTGC
MCM2-F	ATGGCGGAATCATCGGAATCC
MCM2-R	GGTGAGGGCATCAGTACGC
CDH2-F	AGCCAACCTTAACTGAGGAGT
CDH2-R	GGCAAGTTGATTGGAGGGATG
FN1-F	AGGAAGCCGAGGTTTTAACTG
FN1-R	AGGACGCTCATAAGTGTCACC
VIM-F	AGTCCACTGAGTACCGGAGAC
VIM-R	CATTTCACGCATCTGGCGTTC
GAPDH-F	CTGGGCTACACTGAGCACC
GAPDH-R	AAGTGGTCGTTGAGGGCAATG

### Statistical analyses

The experimental data were presented as mean ± SD. GraphPad Prism v8 (GraphPad Software, Inc.) and R (v 4.2) language were used for statistical analysis. Experiments were repeated at least three times. Statistical analysis was performed using one-way or two-way ANOVA. A p-value < 0.05 was considered statistically significant. *P < 0.05, **P < 0.01, ***P < 0.001, ****P < 0.0001.

## Results

### Differential expression of TRPM family genes between tumor and normal group, CMS subtype, and clinical characteristic subgroups

**Figure 1:**
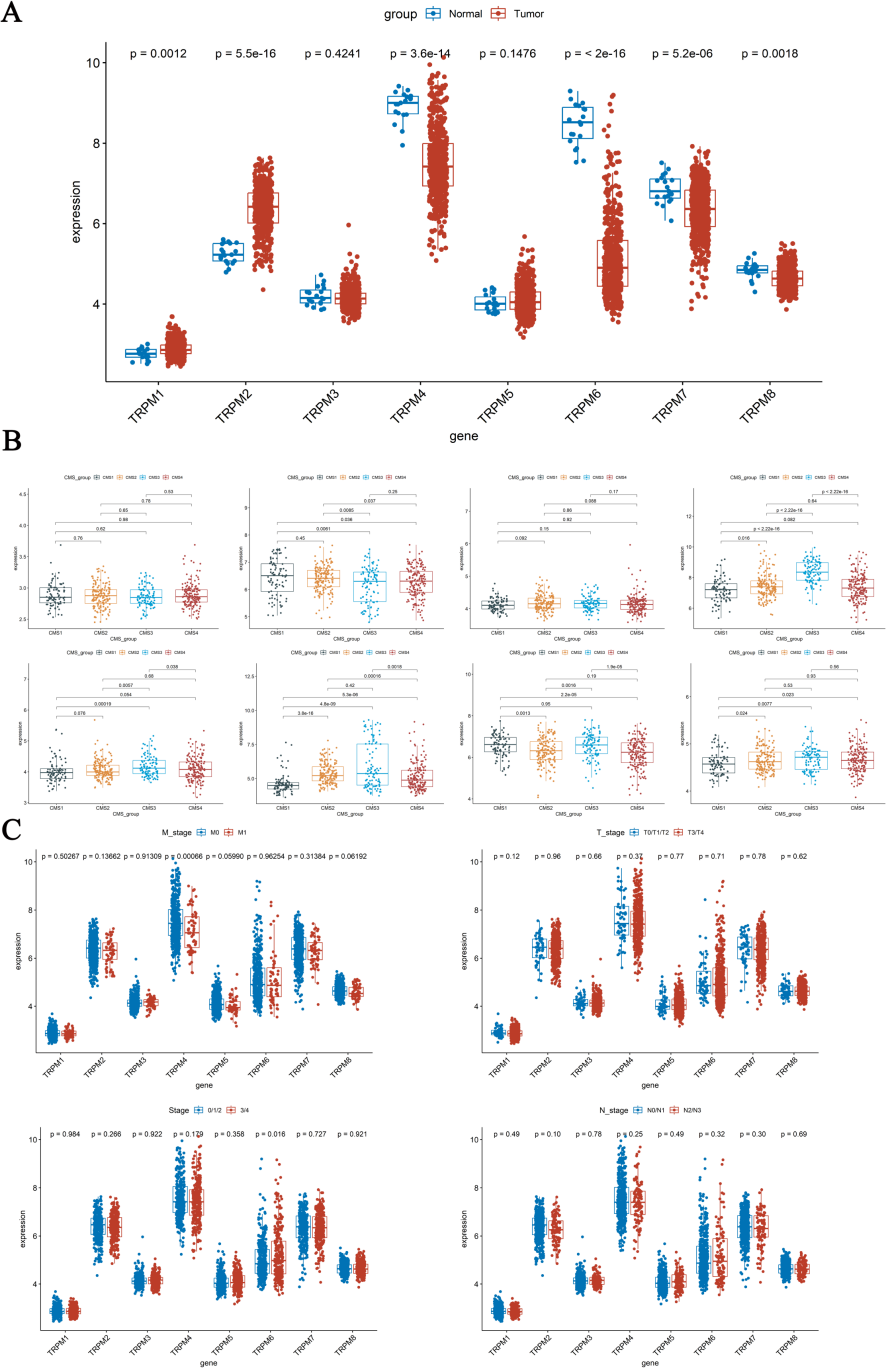
Differential expression of TRPM family genes in COAD **(A)**, Differential expression of TRPM family genes among four CMS subtypes in GSE39582 **(B)**, Differential expression among M stage, T stage, N stage and Stage subgroups in GSE39582 **(C)**.

To characterize the differential expression of TRPM family genes in COAD, our study revealed significant intergroup differences (*P* < 0.05) for TRPM1, TRPM2, TRPM4, TRPM6, TRPM7, and TRPM8 in GSE39582 (**Figure 1A**). Specifically, TRPM1 and TRPM2 were upregulated in tumor tissues, whereas TRPM4, TRPM6, TRPM7, and TRPM8 were downregulated in the tumor group.

The CMS classify the CRC into distinct molecular categories based on comprehensive profiling. The four CMS subtypes show unique biological traits: CMS1 features high immune cell infiltration, CMS2 involves upregulated canonical pathways like WNT signaling, CMS3 is marked by extensive metabolic alterations, and CMS4 exhibits a mesenchymal phenotype with characteristic hallmarks (17). As shown in our study, in both GSE39582 and GSE17536 datasets, significant differential expression was observed for TRPM4 between CMS1 and CMS2, CMS1 and CMS3, CMS2 and CMS3, and CMS3 and CMS4 (*P* < 0.05, **Figure 1B**, **Supplementary Figure 1**). Additionally, TRPM6 between CMS1 and CMS2, CMS1 and CMS3, CMS1 and CMS4, and CMS2 and CMS4 also displayed significant differential expression (*P* < 0.05, **Figure 1B**, **Supplementary Figure 1**). It is particularly noteworthy that TRPM4 different expression in M stage and TRPM6 showed differential expression Stage subgroups in GSE39582 (*P* < 0.05, **Figure 1C**). The expression of TRPM6 in Stage I-II is significantly higher than that in Stage III-IV. The above research results indicated that the TRPM family genes may have different functions in the various subtypes of CMS and Stage subgroups.

### Analysis of the differences in KM survival curves between the high- and low-expression groups

**Figure 2:**
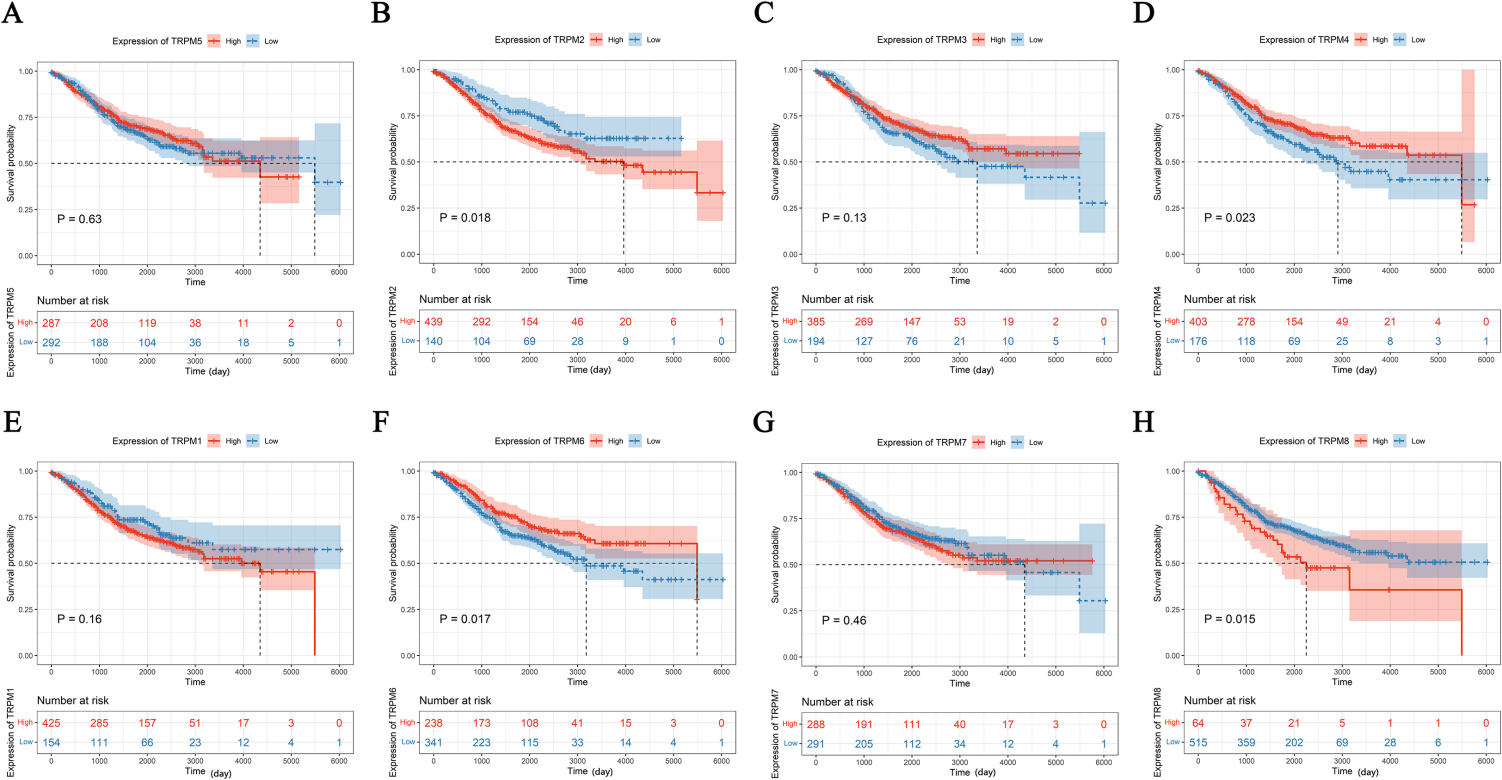
KM survival curves between the high- and low-expression groups of TRPM family genes in GSE39582. TRPM5 **(A)**, TRPM2 **(B)**, TRPM3 **(C)**, TRPM4 **(D)**, TRPM1 **(E)**, TRPM6 **(F)**, TRPM7 **(G)**, TRPM8 **(H)**.

**Figure 3:**
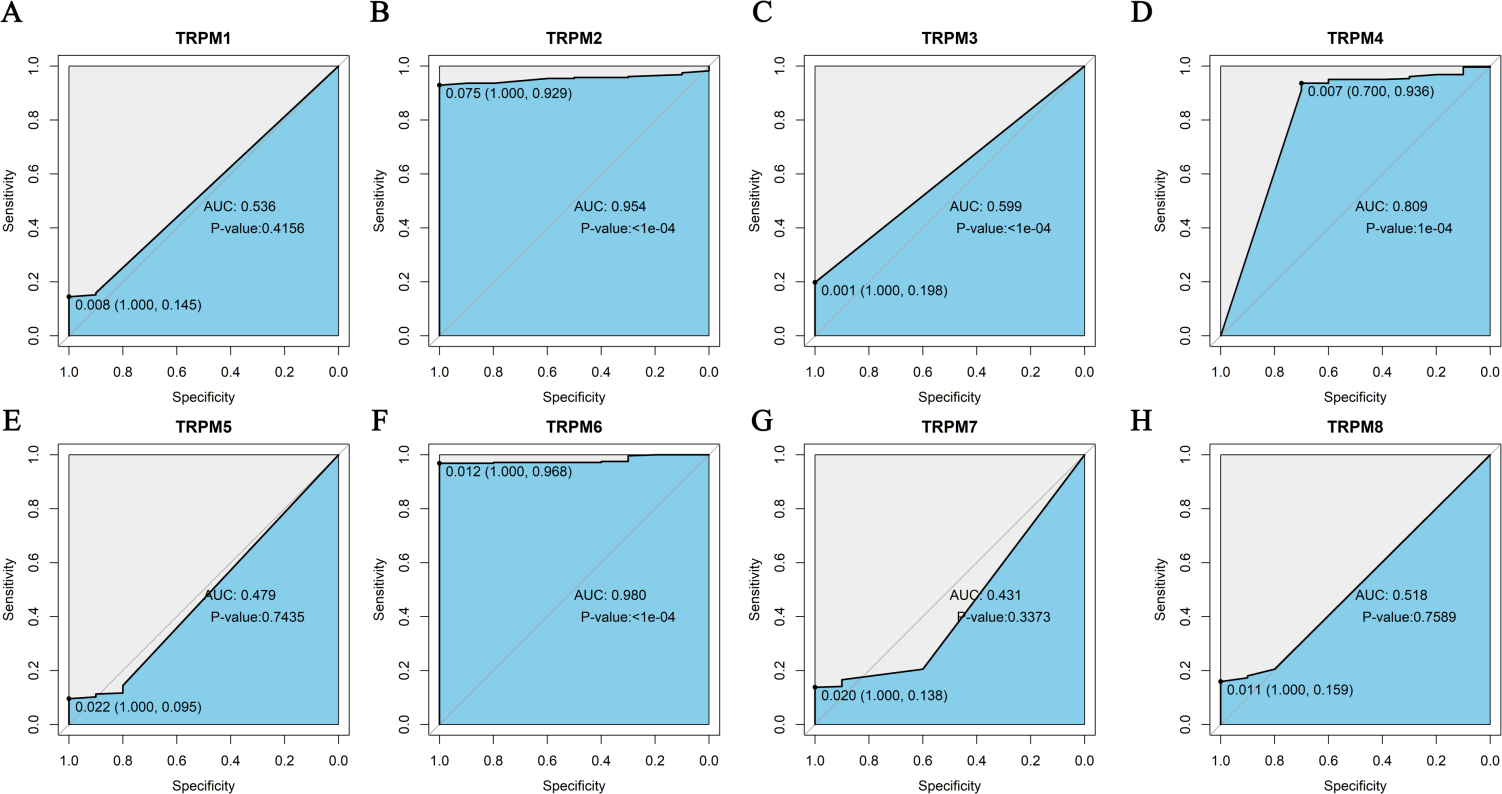
Diagnostic efficiency of TRPM family genes in receiver operating characteristic (ROC) curves. TRPM1 **(A)**, TRPM2 **(B)**, TRPM3 **(C)**, TRPM4 **(D)**, TRPM5 **(E)**, TRPM6 **(F)**, TRPM7 **(G)**, TRPM8 **(H)**.

In the GSE39582, significant disparities were observed for TRPM2, TRPM4, TRPM6, and TRPM8 (*P* < 0.05, **Figure 2**, **Supplementary Figure 2**), where low-expression patients showed improved survival in TRPM2 and TRPM8 (**Figures 2B, H**), whereas high-expression patients had better outcomes in TRPM4 and TRPM6 (**Figures 2D, F**). In the GSE17536, KM survival analysis revealed significant differences between high- and low-expression groups for TRPM3, TRPM4, TRPM6, and TRPM8, with high-expression patients exhibiting better overall survival (*P* < 0.05, **Supplementary Figure 2**). Above all, the high expression of TRPM6 and TRPM4 in COAD is associated with a favorable prognosis for the patients, both had highly diagnostic efficiency in ROC (AUC = 0.809, and AUC = 0.980, respectively, **Figures 3D, F**).

### TRPM6 may regulate highly relevant cancer types by regulation of neural synapses pathways

**Figure 4:**
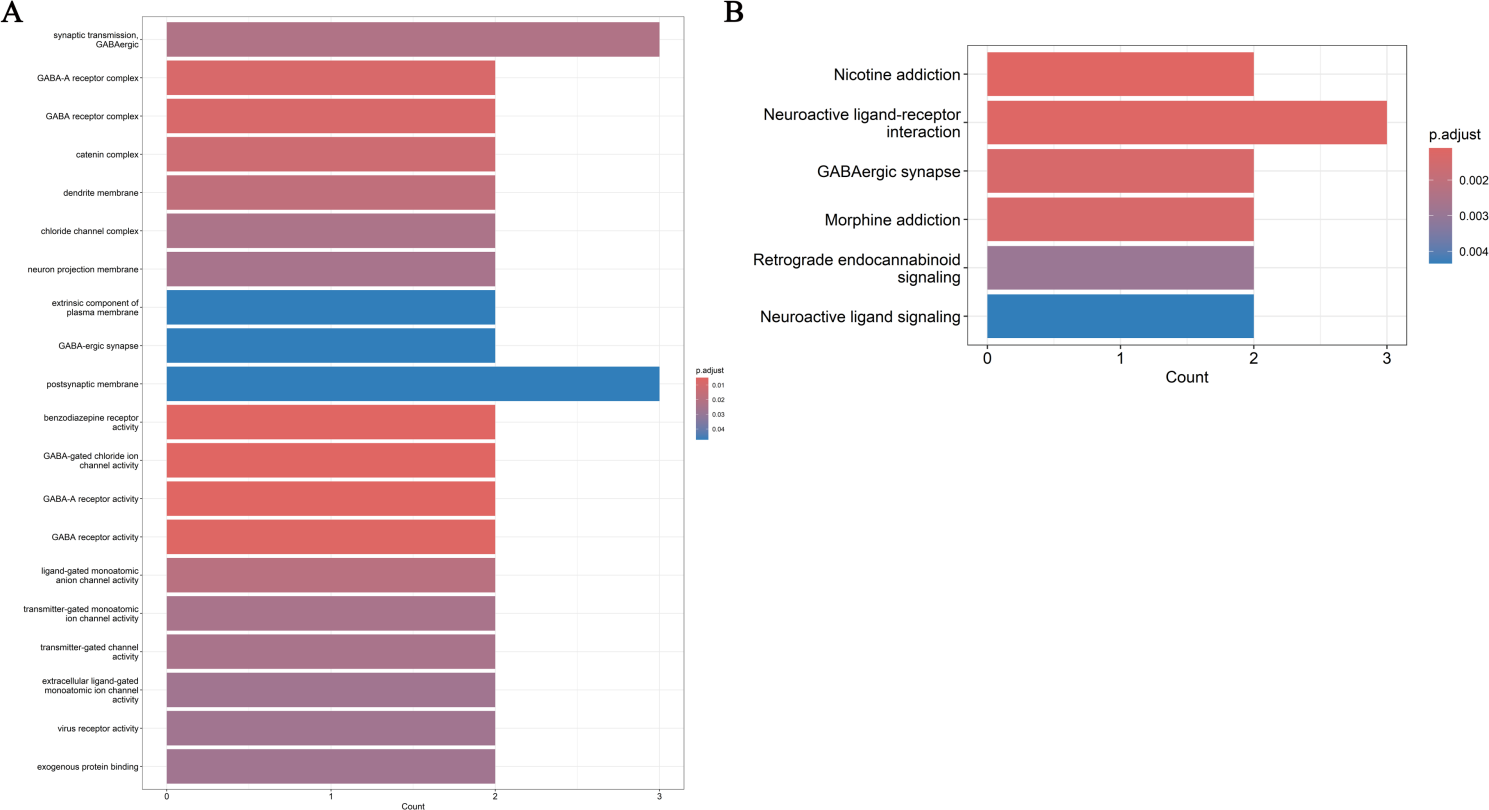
Enrichment analysis in Gene Ontology (GO) **(A)** and Kyoto Encyclopedia of Genes and Genomes (KEGG) **(B)**.

To further investigate whether TRPM6 influences the occurrence and progression of pan-cancer through common regulatory mechanisms, the DEGs in different cancers were shown in **Supplementary Table S1**. We were surprised to discover that TRPM6 played a role in upregulating genes in various types of cancers. Furthermore, we identified 43 significantly regulated genes, mechanistic studies further indicated that these genes predominantly associated with the synaptic transmission-GABAergic, postsynaptic membrane, and neuroactive ligand-receptor interaction signaling pathways, indicating TRPM6 and these regulated genes related to the regulation of neural synapses (**Figures 4A, B**). These results highlight its potential as a therapeutic target for cancer precision medicine.

### Diagnostic value of TRPM6 in COAD

**Figure 5:**
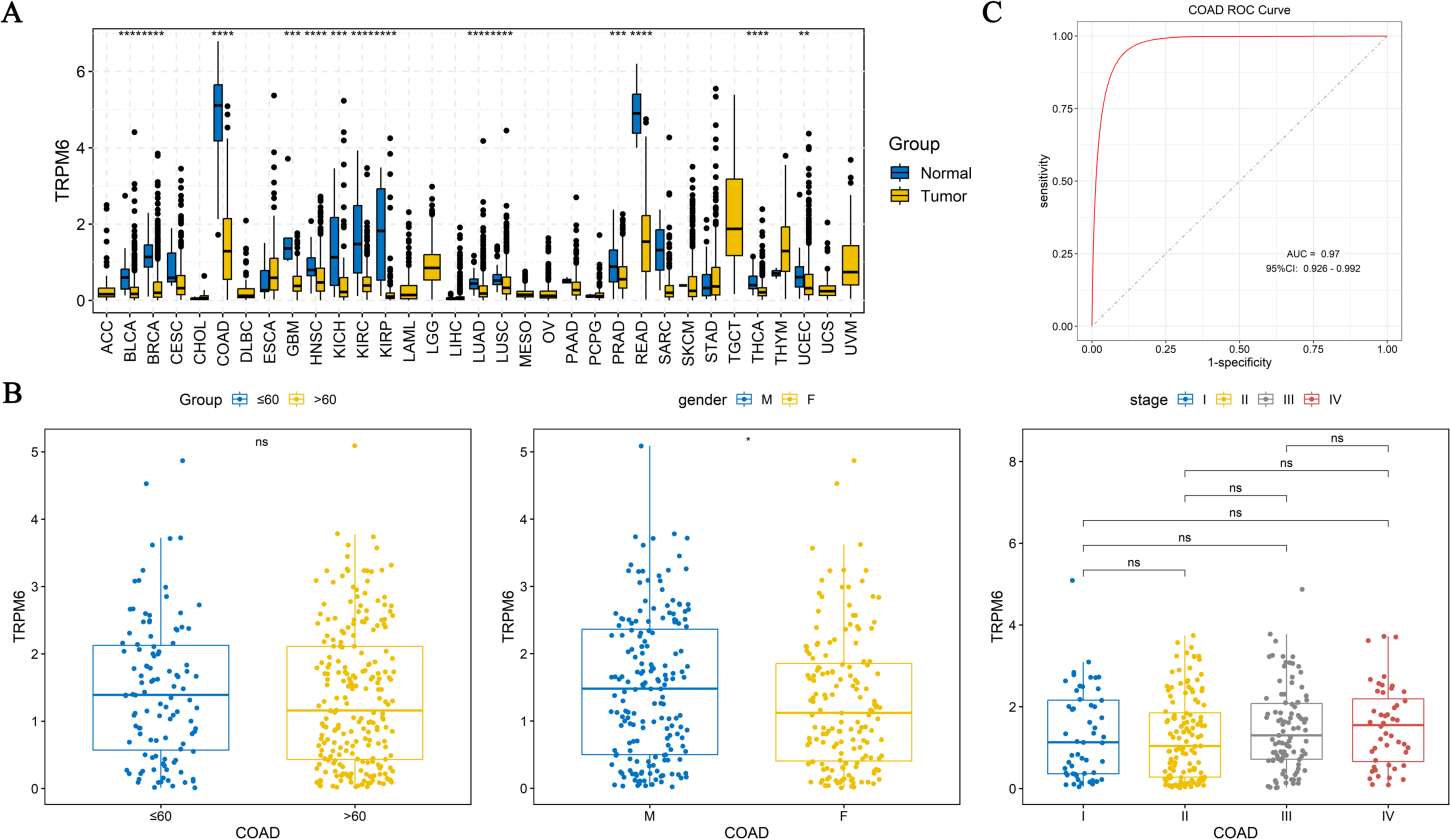
Differential expression of TRPM6 in pan-cancer **(A)**, Differential expression among age, gender, and Stage subgroups of TRPM6 **(B)**, Diagnostic efficiency of TRPM6 **(C)**. “ns” indicates P > 0.05; “*” indicates P < 0.05; “**” indicates P < 0.01; “***” indicates P < 0.001; and “****” indicates P < 0.0001.

Firstly, TRPM6 showed differential expression between tumor and normal tissues in bladder urothelial carcinoma (BLCA), breast invasive carcinoma (BRCA), COAD, glioblastoma multiforme (GBM), head and neck squamous cell carcinoma (HNSC), kidney chromophobe (KICH), kidney renal clear cell carcinoma (KIRC), lung adenocarcinoma (LUAD), lung squamous cell carcinoma (LUSC), prostate adenocarcinoma (PRAD), rectum adenocarcinoma (READ), thyroid carcinoma (THCA), and uterine corpus endometrial carcinoma (UCEC) (**Figure 5A**). Notably, TRPM6 was downregulated in tumor samples of COAD (*P* < 0.05). And there was a differential expression of TRPM6 among the Gender subgroups (*P* < 0.05, **Figure 5B**). What is of concern is that the ROC curve showed that the AUC value = 0.976, indicating that TRPM6 can be used as a reliable diagnostic marker for COAD (**Figure 5C**).

### Analysis of TRPM6 in immune microenvironment

**Figure 6:**
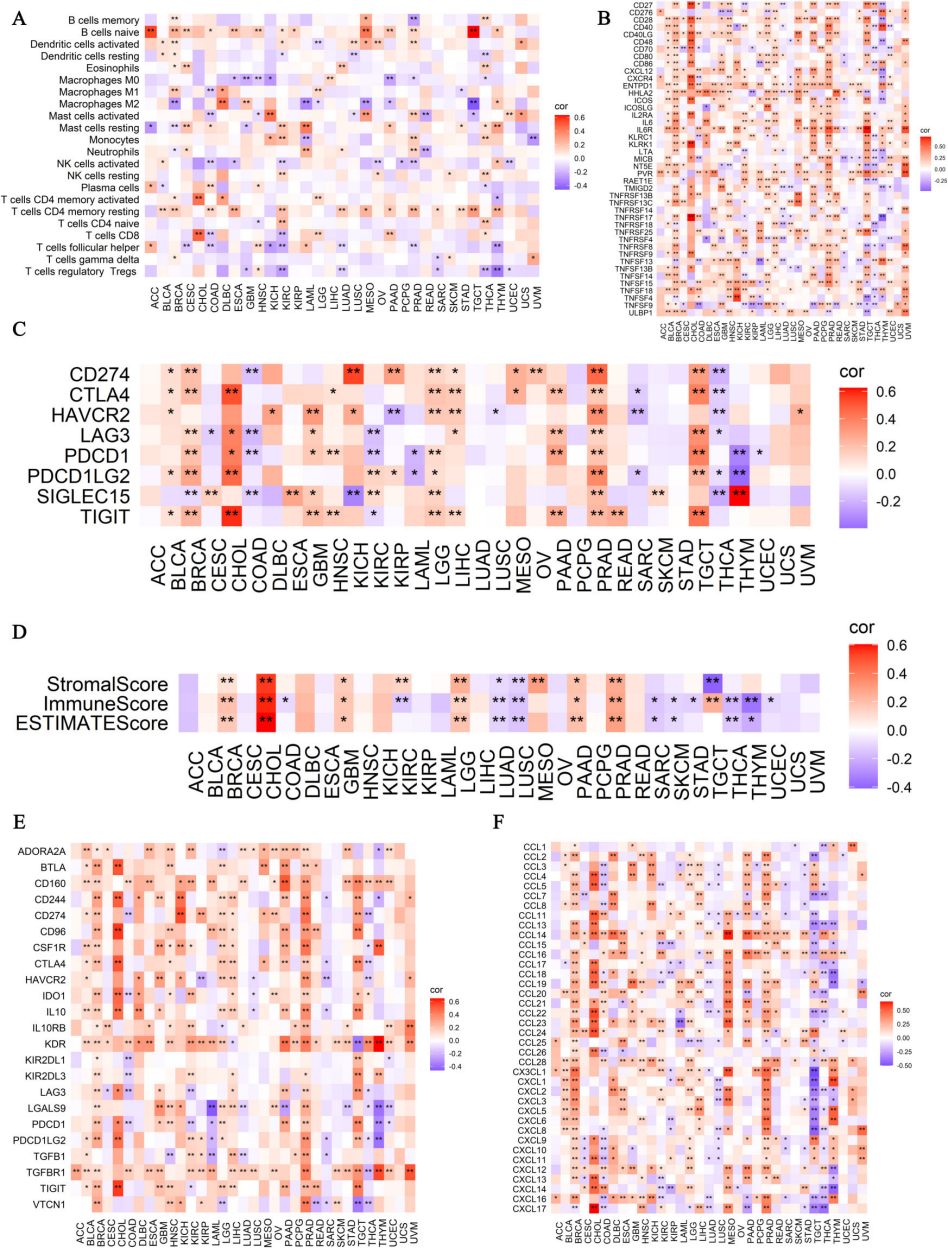
Spearman correlation analysis between TRPM6 and immune cells **(A)**, immunostimulants **(B)**, immunosuppressants **(C)**, immune scores **(D)**, immune checkpoints **(E)**, and chemokines **(F)**. “*” indicates P < 0.05; “**” indicates P < 0.01; “***” indicates P < 0.001; and “****” indicates P < 0.0001.

In adrenocortical carcinoma (ACC) and testicular germ cell tumors (TGCT), TRPM6 exhibited a strong positive correlation with naive B cells (*P* < 0.01). In pan-cancer, TRPM6 showed a robust positive association with resting memory CD4+ T cells (*P* < 0.01, **Figure 6A**). Additionally, TRPM6 displayed a significant negative correlation with TNFSF9 (*P* < 0.01, **Figure 6B**). Among the immune checkpoint inhibitors included in this study, the majority demonstrated positive correlations with TRPM6 across various cancer types (**Figure 6C**). Notably, TRPM6 was strongly and positively correlated with the StromalScore, ImmuneScore, and ESTIMATEScore in cholangiocarcinoma (CHOL) (**Figure 6D**). Conversely, TRPM6 was significantly and negatively correlated with the ImmuneScore in COAD (*P* < 0.05, **Figure 6D**). Furthermore, in pan-cancer, TRPM6 was negatively correlated with CD274, LAG3, PDCD1, and SIGLEC15, and showed significant differences (*P* < 0.01, **Figure 6E**). It had the strongest positive correlation with CXCL14 (*P* < 0.01, **Figure 6F**).

### Analysis of TRPM6 in TMB and MSI

**Figure 7:**
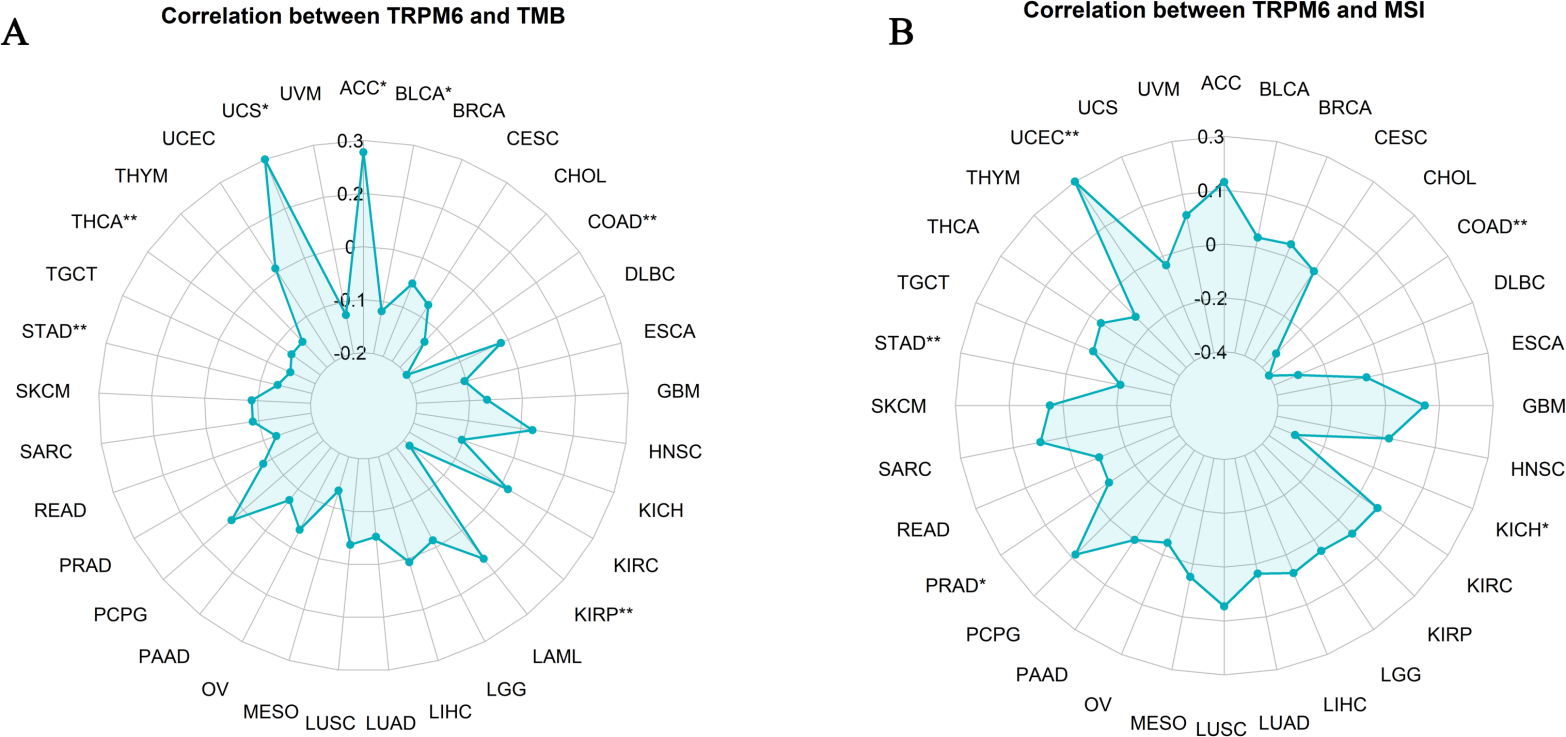
Correlation analysis between TRPM6 and tumor mutation burden (TMB, **A**) and microsatellite instability (MSI, **B**) across pan-cancer.

TRPM6 demonstrated a significant positive correlation with TMB in uterine serous carcinoma (USC) and ACC (*P* < 0.05), whereas a robust negative association was observed in BLCA, COAD, and kidney renal papillary cell carcinoma (KIRP) (*P* < 0.05, **Figure 7A**). Additionally, TRPM6 exhibited a pronounced positive correlation with MSI in UCEC (*P* < 0.01), contrasting with a significant negative correlation in COAD and KICH (*P* < 0.05, **Figure 7B**).

### TRPM6 knockdown promotes CRC cell proliferation and migration capacities

**Figure 8:**
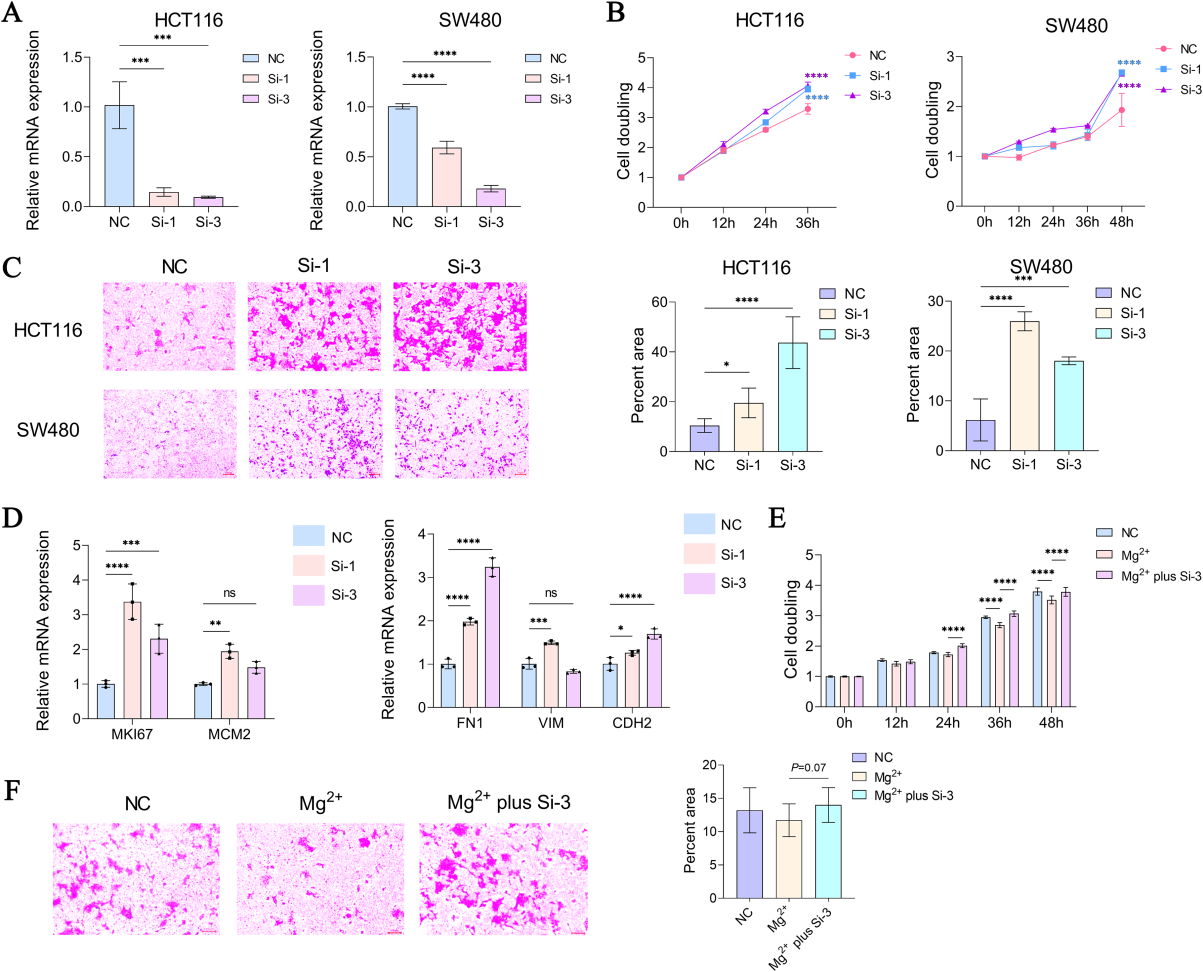
RT-q-PCR validation of knockdown efficiency of two siRNAs targeting TRPM6 in HCT116 and SW480 cells **(A)**, CCK-8 assays in HCT116 and SW480 cells following siRNA knockdown (Two-way ANOVA test, **B**), Comparison of cell migration capacity using Transwell assays (One-way ANOVA test, **C**), RT-q-PCR detection of proliferation and EMT-related markers in HCT116 cells following siRNA transfection **(D)**, Rescue assay of Mg2+ incubation and TRPM6 knockdown (CCK-8, **E**), Rescue assay of Mg2+ incubation and TRPM6 knockdown (Transwell, **F**). NC represents Negative Control siRNA (Non-silencing scrambled siRNA). “*” indicates P < 0.05; “**” indicates P < 0.01; “***” indicates P < 0.001; and “****” indicates P < 0.0001.

To validate the potential tumor-suppressive role of TRPM6, this study employed two colorectal cancer cell lines, HCT116 and SW480, for tumor phenotype analysis. We designed and validated two siRNAs that effectively knocked down TRPM6 expression (**Figure 8A**). CCK-8 assays revealed that TRPM6 knockdown significantly increased the proliferative capacity of HCT116 cells and SW480 cells (**Figure 8B**). To assess the impact of TRPM6 knockdown on cell migration capacity, we conducted Transwell assays. Following 48 hours of incubation, TRPM6 knockdown profoundly promoted cell migratory potential. In HCT116 cells, the proportion of migrated area was elevated by approximately 3-fold in the Si-1 group (P < 0.05) and approximately 6-fold in the Si-3 group (*P* < 0.0001) relative to the NC group. In SW480 cells, the Si-1 group exhibited a roughly 5-fold increase, and the Si-3 group displayed a roughly 3-fold increase in migrated area, with both comparisons reaching statistical significance (*P* < 0.0001) (**Figure 8C**). Concurrently, RT-qPCR analysis revealed that the expression levels of tumor proliferation markers (MCM2 and Ki-67) and epithelial-mesenchymal transition (EMT)-related markers (CDH2, VIM, and FN1) were also significantly elevated following TRPM6 knockdown (**Figure 8D**), consistent with the phenotype assay results.

Previous studies have indicated that elevated concentrations of Mg^2+^ can inhibit tumor proliferation and metastasis. As TRPM6 is a key regulatory molecule of Mg^2+^ channels, its deficiency might impair Mg^2+^ transport and its potential tumor-suppressive effects. We found that under high Mg^2+^ conditions (20 mM), both the proliferation (**Figure 8E**) and migration (**Figure 8F**) capacities of HCT116 cells were suppressed. Notably, in cells subjected to both TRPM6 knockdown and high Mg^2+^ treatment, the tumor-suppressive effects mediated by Mg^2+^ were significantly attenuated. This confirms the critical role of TRPM6 in mediating the functional effects of Mg^2+^. In summary, through tumor phenotype experiments, this study provides preliminary evidence that TRPM6 deficiency promotes malignant progression in cancer cells. This lays the groundwork for investigating its role in early tumor warning and the molecular mechanisms by which it regulates Mg^2+^.

## Discussion

COAD exhibits a rising incidence and ranks among the most prevalent malignant tumors (18, 19). TRPM family genes have been implicated as key players in colon-related chemoresistance (20). This study utilized public databases and bioinformatics approaches to investigate TRPM family genes, revealing that most members exhibit significant intergroup differences in COAD and across distinct CMS subtypes (*P* < 0.05). Notably, TRPM4 and TRPM6 showed differential expression across M stages and Stage subgroups, respectively, with high expression of both TRPM6 and TRPM4 associated with favorable prognosis in COAD (*P* < 0.05). Subsequent pan-cancer analyses indicated that TRPM6 may regulate highly relevant cancer types through modulation of neural synaptic pathways and displays high diagnostic value in COAD. Similarly, TRPM6 demonstrated intimate associations with both the immune and tumor microenvironment, suggesting its potential role as a regulator and a candidate biomarker for tumor immunobiology. The results of the tumor phenotype experiments indicated that the knockdown of TRPM6 would promote the proliferation and migration of colon cancer cells (HCT116 and SW480) by up-regulating the expression of proliferation-related and EMT-related markers, and weaken the magnesium ion-mediated tumor suppressive effect. This provided preliminary evidence for the role of TRPM6 deficiency in promoting the malignant progression of colon cancer.

Numerous studies have demonstrated that genes belonging to the TRPM family are involved in tumor initiation and progression (21). The TRPM4 channel, which is permeable to monovalent cations, is predominantly expressed in the intestine and prostate (22, 23). Additionally, this channel is expressed in cells of the innate and adaptive immune responses (24–26). TRPM4 participates in various physiological processes, including T cell activation and the migration of endothelial cells and certain immune cells (27). In most studies, TRPM4 protein expression levels are reported to be elevated in tumor samples compared to healthy tissues (10, 28–30). However, in urinary bladder cancer, no such changes at the protein or mRNA level have been documented (31). For colorectal cancer, TRPM4 mRNA expression has been described as either decreased or unchanged relative to control tissues (21, 32). Notably, downregulation of the TRPM6 channel is observed in 80% of primary colorectal cancer tumors (13, 33), and the findings of the present study further validate these previous results. Interestingly, most investigations into TRPM4 and TRPM6 expression in cancer have reported alterations in their expression levels (34, 35). However, to date, no associations of TRPM4 or TRPM6 with CMS subtypes have been identified. Our research addresses these gaps in the field, extending to analyses of M stages and stage subgroups. Furthermore, Kaplan-Meier survival analysis revealed that high expression of TRPM6 and TRPM4 correlates with better overall survival in patients, indicating a favorable prognosis for COAD patients (36). These findings not only enhance our understanding of the role of TRPM4 and TRPM6 in colorectal cancer heterogeneity, particularly their potential as subtype-specific and stage-associated biomarkers, but also lay a foundation for future studies exploring their mechanistic involvement in CMS subtype differentiation and disease progression. Given their association with favorable prognosis, TRPM4 and TRPM6 may represent promising targets for prognostic stratification and the development of tailored therapeutic strategies in CRC management. Although our study establishes TRPM4 as a robust prognostic biomarker, the detailed molecular mechanisms underlying its function in COAD remain to be elucidated and represent an important area for future investigation.

Further pan-cancer research findings have revealed that TRPM6 functions to upregulate genes across various cancer types, which is consistent with the aforementioned study results. Additionally, to explore the pathways regulated by TRPM6, enrichment analysis indicated that TRPM6 may modulate highly relevant cancer types through the regulation of neural synaptic pathways. Neuronal activity is well established as a key regulator of neurodevelopment and plasticity, and it is increasingly recognized as a critical factor in driving cancer growth (37, 38). Given that TRPM6 regulates neural synaptic pathways, it is plausible that TRPM6 may influence COAD development or aggressiveness through similar neuro-cancer cross-talk. It is tempting to speculate that the regulation of neural synaptic pathways by TRPM6 might, in turn, contribute to shaping the unique immune microenvironment we observed, perhaps through the release of neurotransmitters that act on immune cells. Therefore, we propose that intervention targeting the TRPM6 - neural signaling axis may be a new strategy for treating colorectal adenomatous polyps. Future research should focus on verifying this axis and exploring whether regulating the activity of neurons in the tumor microenvironment can affect the development of cancer.

Notably, TRPM6 exhibited a strong positive correlation with naive B cells in ACC and TGCT. Naive B cells are critical for initiating adaptive immune responses through antibody production and antigen presentation (39). They may interact with TRPM6 to modulate humoral immunity in these malignancies, suggesting a potential role for TRPM6 in priming anti-tumor immune surveillance. Conversely, TRPM6 showed a robust positive association with resting memory CD4**+** T cells. These cells are poised to re-activate upon antigen re-encounter (40). This implies that TRPM6 might contribute to maintaining immune memory pools, potentially influencing long-term anti-tumor responses across diverse cancer types. The significant negative correlation between TRPM6 and TNFSF9 adds another layer of complexity. TNFSF9 is a pro-inflammatory ligand involved in T cell activation and macrophage polarization (41). Its downregulation in the presence of high TRPM6 could indicate a regulatory mechanism to temper excessive inflammation. TRPM6-high tumors exhibit a distinct immune phenotype characterized by reduced expression of immune checkpoint molecules (CD274/PD-L1, TNFSF9) and increased infiltration of resting memory T cells. This pattern aligns with an immune quiescent state where the low abundance of PD-L1/TNFSF9 may weaken immunosuppressive signaling (42), while the accumulation of resting memory T cells suggests a reservoir of antigen-experienced T cells that could be reactivated (43). Such a phenotype implies TRPM6 may serve as a potential marker to identify tumors with latent immune responsiveness, providing a rationale for combining TRPM6 targeting with immunotherapies to enhance anti-tumor immunity.

TMB and MSI, as core biomarkers of genomic instability, are closely implicated in tumor immunogenicity and response to immunotherapy (44). At the TMB level, TRPM6 exhibited a significant positive correlation in USC and ACC, whereas a marked negative association was observed in BLCA, COAD, and KIRP. Mechanistically, as a magnesium transporter, TRPM6 may regulate TMB levels through influencing magnesium-dependent DNA repair enzymes (45). Regarding MSI, TRPM6 showed a significant positive correlation in UCEC, in contrast to a significant negative correlation in COAD and KICH. This differential association may be attributed to TRPM6-mediated regulation of MMR function via magnesium homeostasis (46). TRPM6 could potentially inhibit MMR activity in UCEC, while exerting a protective effect on MMR function in COAD and KICH. The present study identified significant cancer-type specificity in the correlations between TRPM6 and TMB/MSI, providing novel insights into the potential role of TRPM6 in genomic regulation and immune microenvironment modulation.

Magnesium (Mg^2+^) is essential for energy metabolism, muscle contraction, and neurotransmission (47, 48). In the colon, transcellular Mg^2+^ absorption is facilitated by TRPM6 (47, 49). Numerous previous clinical studies have highlighted the potential benefits of Mg in the prevention and treatment of CRC (50), while Mg supplementation has been shown to enhance therapeutic efficacy and mitigate severe side effects in CRC patients. Meanwhile, the EMT regulatory network in CRC is highly complex, representing a cellular reprogramming event that occurs during embryonic development as well as in the maintenance of adult tissue homeostasis (51). Consistent with these observations, our *in vitro* findings, which establish TRPM6 as a critical mediator of Mg^2+^-dependent tumor suppression, provide a mechanistic explanation for the clinically observed protective role of magnesium in COAD. These results align with prior clinical evidence supporting Mg’s protective role in CRC and further clarify that TRPM6 functions as a critical mediator of Mg^2+^-dependent tumor suppression, emphasizing the importance of functional TRPM6 in enabling Mg^2+^ to exert its beneficial effects in colorectal carcinogenesis. As the upstream initiating event, TRPM6 mediates Mg^2+^ influx into tumor and stromal cells—Mg^2+^, as a cofactor for multiple enzymes, first regulates neuro pathway-related genes by enhancing their transcriptional activity or stabilizing mRNA, thereby modulating neural synaptic pathways signaling. Concurrently, Mg^2+^ influx reshapes the immune microenvironment: it downregulates immunosuppressive molecules (e.g., CD274/PD-L1) and upregulates resting memory T cell infiltration, while inhibiting pro-tumor immune subsets. These neuro-modulatory and immune-regulatory effects synergistically suppress tumor cell proliferation, invasion, and angiogenesis, ultimately restraining tumor progression.

### Limitation

This study has several limitations. First, the functional validation of TRPM6 and TRPM4 was restricted to two CRC cell lines (HCT116 and SW480), and *in vivo* animal models are needed to confirm their roles in tumor progression. Second, the molecular mechanisms underlying TRPM6-mediated regulation of neural synaptic pathways and Mg^2+^ transport in CRC remain incompletely characterized, requiring further mechanistic studies. Third, the associations between TRPM family genes and CMS subtypes, TMB, and MSI were derived from bioinformatics analyses of public datasets, necessitating independent clinical cohorts to verify these findings.

## Conclusion

In summary, this study identifies TRPM4 and TRPM6 as key prognostic biomarkers in COAD. Furthermore, we provide a multi-faceted mechanistic elucidation of TRPM6’s role in immune microenvironment, thus making TRPM6 a potential biomarker for predicting immune therapy response. Mechanistically, in terms of mechanism, our data suggest that TRPM6 may regulate cancer progression, possibly through neural synaptic pathways, modulates the immune microenvironment, and mediates Mg^2+^-dependent tumor suppression, with its knockdown promoting CRC cell proliferation, migration, and EMT marker upregulation while attenuating Mg^2+^-mediated anti-tumor effects. These findings highlight TRPM6 as a critical mediator of Mg^2+^ function and a promising candidate for prognostic stratification and therapeutic targeting in CRC, laying the groundwork for future studies on its regulatory mechanisms in tumor progression and the development of Mg^2+^-based therapeutic strategies. Meanwhile, our *in vitro* experiments provide a theoretical basis for the ‘precision magnesium supplementation’ strategy, i.e., future clinical trials could be designed to evaluate the adjuvant therapeutic effect of Mg^2+^ supplements in patients with high TRPM6 expression.

## Data Availability

The datasets presented in this study can be found in online repositories. The names of the repository/repositories and accession number(s) can be found in the article/**Supplementary Material**.

## Supplementary material

Supplementary Figure 1 Differential expression of TRPM family genes among four CMS subtypes in GSE17536. “ns” indicates P > 0.05; “*” indicates P < 0.05; “**” indicates P < 0.01; “***” indicates P < 0.001; and “****” indicates P < 0.0001.

Supplementary Figure 2 KM survival curves between the high- and low-expression groups of TRPM family genes in GSE17536.

Supplementary Table 1 Differentially expressed genes (DEGs) in different cancers.
