# Supplementary figures and images for: TRPM6 acts as a prognostic biomarker and mediates Mg2+ dependent tumor suppression in colon adenocarcinoma
Source: Front Immunol. 2026 Jan 5;16:1686461. doi: 10.3389/fimmu.2025.1686461 (PMC12813124; doi:10.3389/fimmu.2025.1686461)

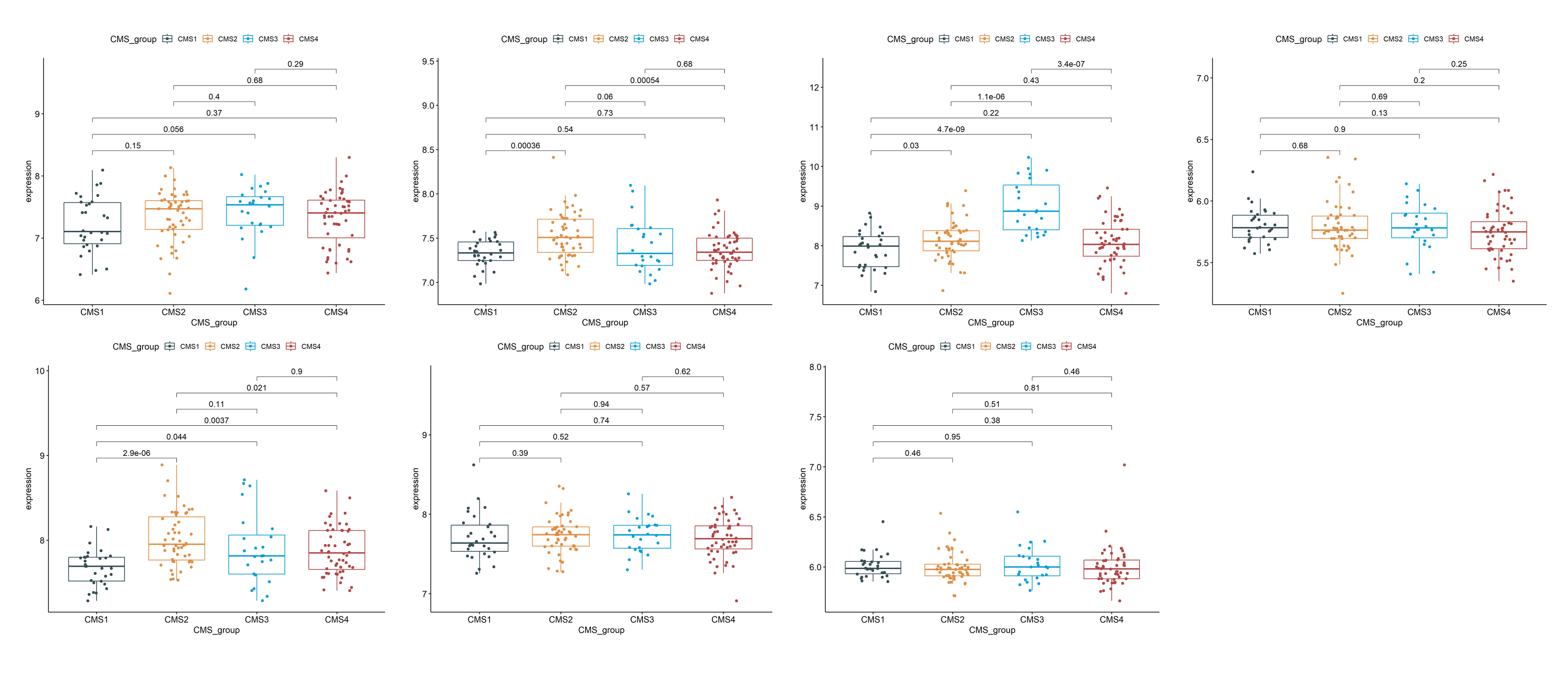

Supplement: Supplementary Figure 1 — Differential expression of TRPM family genes among four CMS subtypes in GSE17536. “ns” indicates P > 0.05; “*” indicates P < 0.05; “**” indicates P < 0.01; “***” indicates P < 0.001; and “****” indicates P < 0.0001. [file Image1.tiff]

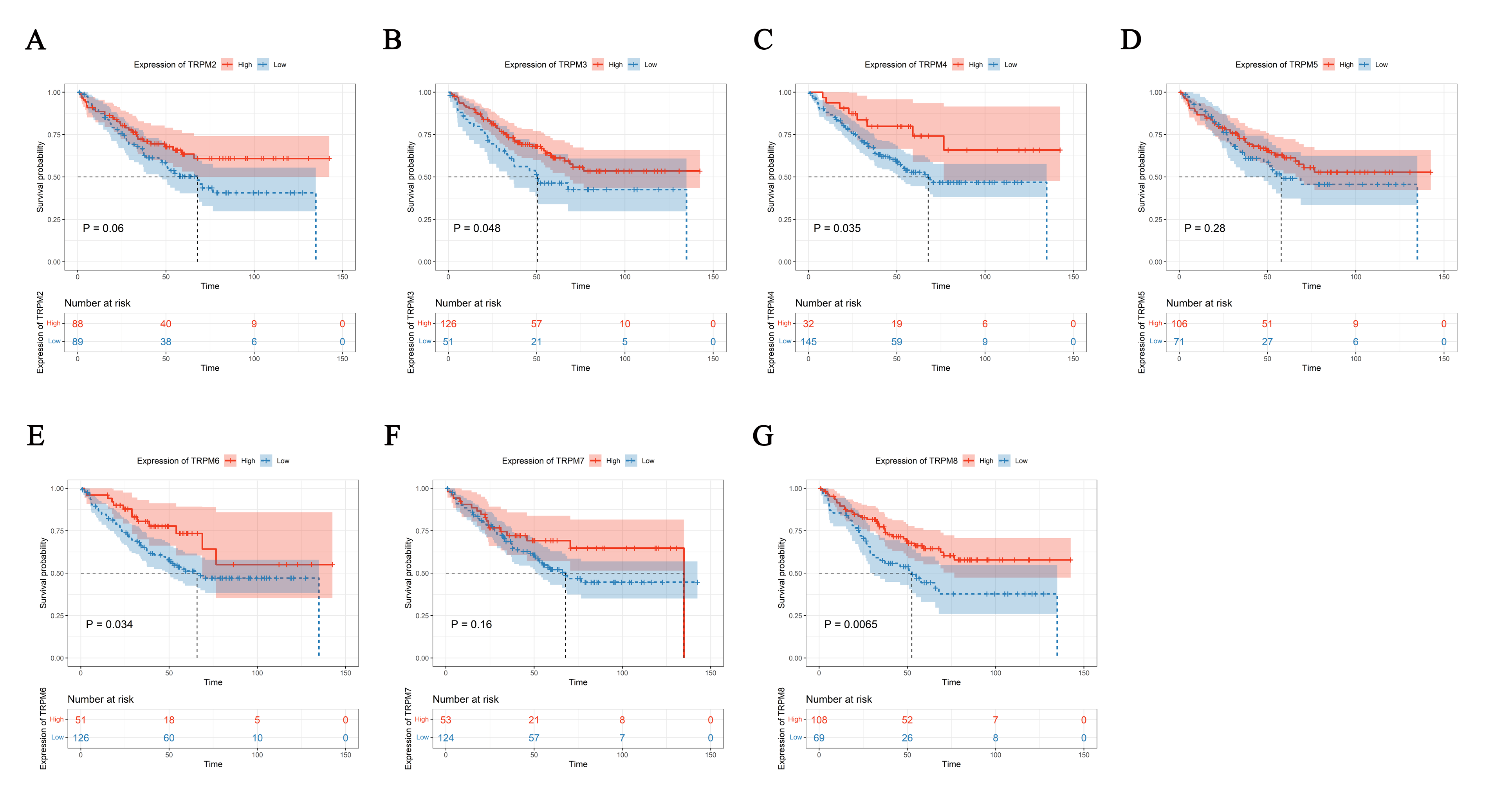

Supplement: Supplementary Figure 2 — KM survival curves between the high- and low-expression groups of TRPM family genes in GSE17536. [file Image2.tiff]
